# Analysis of Skin Microbiome in Facial and Back Acne Patients Based on High‐Throughput Sequencing

**DOI:** 10.1111/jocd.70259

**Published:** 2025-06-12

**Authors:** YiJie Du, BenYue Li, Jie Yang, YeXiang Zhang, FengWei Qi, Hong Meng

**Affiliations:** ^1^ School of Light Industry Science and Engineering Beijing Technology & Business University Beijing China; ^2^ Shandong Huawutang Biotechnology Co., Ltd Shandong China; ^3^ International School of Cosmetics, School of Light Industry Science and Engineering Beijing Technology & Business University Beijing China

**Keywords:** facial and back acne, high‐throughput sequencing, microbial diversity, skin microbiota

## Abstract

**Objective:**

To study the composition and diversity of microorganisms in skin lesions of patients with facial acne and back acne, analyze the relationship between microorganisms and different parts of acne.

**Methods:**

A total of 30 facial acne patients, 30 back acne patients, and 30 healthy controls were included. Comprehensive data on demographics, skin conditions, and state of life were collected. Skin microbiota samples were obtained using standardized protocols, through high‐throughput microbial sequencing and bioinformatics analysis, to assess microbiota diversity, composition, and function, as well as their correlations with skin characteristics.

**Results:**

The diversity of skin microbiota of back acne patients decreased significantly, and the abundance of *p_actinomycetes* and *o_propionate* bacteria increased significantly. They are closely related to glucose metabolism, which may lead to increased trans epidermal water loss, decreased skin moisture content, and increased skin oil content.

**Conclusion:**

The skin microbiota diversity of patients with back acne is low, and high‐abundance bacteria are closely related to glucose metabolism, which may lead to the breakdown of the skin barrier and increased sebum secretion.

## 1 Introduction

Acne is a widespread dermatological issue, particularly prevalent among adolescents and young adults. Epidemiological research indicates that 80%–90% of adolescents experience acne, impacting 9.4% of the global population and ranking as the eighth most common disease worldwide [1]. The incidence of acne has been increasing due to shifts in climate, environment, and lifestyle. The etiology of acne is multifactorial, encompassing endocrine activity, abnormal sebum secretion, incomplete follicular keratinization, and bacterial infection. Notably, the homeostasis of sebaceous glands is intricately linked to acne formation. Increased sebum secretion, alterations in sebum composition, and the development of funnel cysts are hallmark pathological features of acne [2]. Furthermore, excessive keratinization of follicular epithelium, disruption of microecological balance, and inflammatory responses are critical pathological mechanisms contributing to the development of acne [3].

Recent research on skin microbiota has offered a novel perspective on the understanding of acne. Theoretically, the skin environment should be inhospitable to the proliferation of microorganisms. Acting as the body's primary defense against pathogens, the skin secretes sweat that can acidify the skin surface, while skin cells release antibacterial peptides and other antimicrobial compounds. However, in actuality, hair follicles, sebaceous glands, sweat glands, and other skin appendages offer numerous niches for microbial colonization, thereby contributing to the formation of a relatively stable skin microbiota [4]. Consequently, the skin represents the second largest microecological system in the human body, following the gut. A study conducted by the Human Microbiome Project Alliance has revealed that the adult skin microbiota comprises approximately 40 trillion species, representing 16% of the total symbiotic microorganisms within the human body [5]. The advancement of sequencing technologies has facilitated an increasing number of studies, which collectively underscore the significant role of skin microbiota in regulating the physiological microenvironment of the skin, mediating inflammatory and immune responses, and its close association with the pathogenesis and progression of various skin diseases [6, 7].

Currently, several studies have investigated the characteristics of skin microbiota in relation to acne. A study conducted in China examined the variations in skin microbiota among acne patients with varying severity of the condition, as well as in comparison to healthy individuals. The findings revealed significant alterations in the skin microbiota of acne patients relative to the healthy control group. The skin microbiome of patients with grade 1–3 acne exhibits considerable similarity. However, in comparison to these patients, those with grade 4 acne demonstrate a markedly distinct skin microbiome. This distinction is characterized by increased alpha diversity and a higher prevalence of four Gram‐negative bacteria: fecal bacteria, Klebsiella, acidophilic bacteria, and Pseudomonas [8]. A study conducted in South Korea characterized the bacterial and fungal communities in individuals with acne, revealing a higher proportion of *Bacillus subtilis* and *Staphylococcus aureus* in acne patients compared to a healthy control group [9]. Concurrently, another study in the same year investigated the role of skin microorganisms within the hair follicle sebaceous gland unit in the progression of non‐inflammatory acne lesions. The study identified that Malassezia and *Staphylococcus epidermidis* may significantly contribute to the pathogenesis of inflammatory lesions in acne patients, paralleling the role of *Propionibacterium acnes* [10]. While certain studies have examined the composition of the microbiota associated with acne‐affected skin, comparative investigations into the disparities between facial and back acne microbiota remain limited. Considering the physiological distinctions between the facial and back regions, it is plausible that their microbial compositions exhibit substantial differences. Furthermore, elucidating these differences could serve as a crucial foundation for the development of precise, targeted therapeutic strategies.

## 2 Methods

### 2.1 Study Subjects

This research enlisted a cohort of 90 participants, aged 18–29, from the Beijing region during the period from April to June 2024. The cohort was composed of three groups: 30 individuals with facial acne, 30 individuals with back acne, and a control group of 30 individuals, with each group maintaining an equal distribution of genders.

#### 2.1.1 Inclusion Criteria

Participants were included if they: met the diagnostic criteria for acne, were aged between 18 and 29 years, and could understand and voluntarily sign the informed consent form.

#### 2.1.2 Exclusion Criteria

Participants were excluded if they: had concurrent facial eczema, atopic dermatitis, rosacea, or other skin diseases; were pregnant or lactating; had used systemic antibiotics, corticosteroids, retinoids, or immunosuppressants within the past month; had used topical antibiotics, corticosteroids, or retinoids within the last 7 days; refused to sign the informed consent form.

### 2.2 Data and Sample Collection

#### 2.2.1 Collection of Clinical Information

Participants completed a questionnaire recording general information and scales for depression and quality of life. Non‐invasive assessments and dermatological imaging techniques were employed to evaluate the severity of acne, skin moisture levels, barrier function, sebum content, and skin pH.

#### 2.2.2 Collection of Skin Microbiota Samples

Participants abstained from bathing for 24 h and avoided washing their faces or using skincare products for 8 h prior to sample collection. Samples were collected from cheek skin lesions using sterile cotton swabs moistened with saline solution. Samples were stored in cryotubes and frozen in liquid nitrogen at −80°C.

### 2.3 Skin Microbiota 16S Ribosomal Ribonucleic Acid (rRNA) Sequencing Process

The genomic deoxyribonucleic acid (DNA) from the samples was extracted using the OMEGA Mag‐bind soil DNA kit, and the purity and concentration of the DNA were evaluated. Specific primers with barcodes were used along with high‐fidelity DNA polymerase to amplify the selected V3–V4 variable region through Polymerase Chain Reaction (PCR) according to the chosen sequencing region. The amplification reaction system for PCR. The PCR reaction parameters were set as follows: a pre‐denaturation step at 94°C for 3 min, followed by a denaturation phase at 94°C for 5 s, an annealing step at 57°C for 90 s, an extension phase at 72°C for 10 s, and a final extension step at 72°C for 5 min. This cycle was repeated for a total of 24 iterations. The PCR products were analyzed using 2% agarose gel electrophoresis, and the target fragments were excised and recovered from the gel. Gel recovery was performed using the Quant‐iT PicoGreen dsDNA Assay Kit. Based on the preliminary quantification results from electrophoresis, the PCR amplification recovery products were quantified using a Microplate reader (BioTek, FLx800) fluorescence quantification system. The products were mixed in appropriate proportions based on the sequencing requirements for each sample.

Library construction was carried out using the TruSeq Nano DNA LT Library Prep Kit from Illumina. The constructed libraries underwent quality control using the Agilent Bioanalyzer 2100 and Promega QuantiFluor assays. Once the library quality was confirmed, the samples were subjected to sequencing.

### 2.4 Statistical Analysis

The sample data were analyzed using Statistical Package for the Social Sciences (SPSS) 25.0 statistical software. Quantitative data following a normal distribution were expressed as mean ± standard deviation (x ± s). Two‐group and multiple‐group comparisons were conducted using t‐tests and analysis of variance (ANOVA), respectively. Qualitative data were analyzed using chi‐square tests. Alpha diversity between groups was compared using the Wilcox Test function for two groups and the Kruskal Test function for three groups or more. Differences in community structures between groups were compared using the PERMANOVA function. In this study, *p* < 0.05 indicates statistical significance, and *p* < 0.01 indicates significance.

### 2.5 Bioinformatics Analysis

#### 2.5.1 Sequencing Data Processing

The initial dataset is presented in FASTQ format. Following data processing, the Cutadapt software is initially used to trim primer sequences from the raw data. Subsequently, DADA2 is employed to perform quality filtering, denoising, merging, and chimera removal on the qualified paired‐end raw data according to the default parameters of QIIME 2, resulting in representative sequences and an ASV abundance table.

#### 2.5.2 Description of Sequencing Results

##### 2.5.2.1 Operational Taxonomic Unit (OTU) Count

The Venn diagram effectively illustrates the distribution of OTUs across individual samples, highlights the overlap of OTUs among various sample groups, and quantifies the shared OTU count.

##### 2.5.2.2 Taxonomic Analysis

To assess the adequacy of sample sizes in this study, a species accumulation curve can be constructed. The *x*‐axis of the species accumulation curve represents sample number, while the *y*‐axis represents the OTU count. By increasing the sample size, the trend of OTUs can be observed. In cases of limited sample sizes, the discovered species may not be comprehensive. As the sample size increases, a sharp upward trend in the curve indicates the discovery of numerous new species. If this trend continues at the end of the curve, it suggests an insufficient sample size, with the potential to discover more new species by increasing sample size. If the curve flattens at the end, it signifies that increasing the sample size will not yield more new species, indicating that the current sample size adequately reflects the species composition of the community.

##### 2.5.2.3 Abundance Rank Curve

Studying the abundance rank curve allows for a better understanding of sample diversity, including richness and evenness. The length of the *x*‐axis can be used to infer the richness of species in the sample, with a positive correlation between the two. The shape of the curve along the *y*‐axis clearly illustrates the distribution of species in the sample; a flatter curve indicates a more even distribution of species.

#### 2.5.3 Community Diversity Analysis

Using the Qiime2 Software to Calculate α and β Diversity Indices.

Alpha diversity indices are utilized to quantify species richness and evenness within a given sample. Indices such as Abundance‐based Coverage Estimator (ACE) and Chao specifically evaluate species richness, whereas the Shannon and Simpson indices provide insights into both species richness and evenness within the sample. The Kruskal‐Wallis test is employed to assess differences in alpha diversity indices across multiple groups.

Beta diversity indices are utilized to compare and analyze the composition of microbial communities across various samples. Due to the vast number of microbes in samples and the multidimensional differences between microbes in different samples, direct comparisons are challenging. Therefore, Beta diversity is employed to measure the similarity of microbes between different samples. By calculating distance matrices such as UniFrac distance and Bray‐Curtis distance, Beta diversity assesses the evolutionary levels of different sample sequences, providing a better understanding of microbial community differences. UniFrac distance measures differences in OTUs between different communities, revealing their similarities. UniFrac distance can be categorized into Unweighted UniFrac distance, which only reflects the presence or absence of species in a sample, and Weighted UniFrac distance, which considers both the presence or absence of species and their abundance in a sample. After obtaining distance matrices for each sample, Principal Coordinates Analysis (PCoA) and Nonmetric Multidimensional Scaling (NMDS) can be used to reduce multidimensional data, visually demonstrating the most important elements and structures.

#### 2.5.4 Inter‐Group Difference Analysis

Linear discriminant analysis Effect Size (LEfSe) can be used to identify species features that best explain inter‐group differences in two or more sets of samples, as well as the extent to which these features influence inter‐group differences. By employing inter‐group difference analysis methods and utilizing rigorous statistical techniques based on obtained community abundance data, species between two or multiple sets of samples can undergo hypothesis testing to evaluate the significance of species abundance differences, thereby obtaining information on species with significant differences between the groups. The Statistical Analysis of Metagenomic Profiles (STAMP) difference analysis uses the Kruskal–Wallis test to compare species abundance between groups of samples. Through this analysis, significant species differences can be identified.

#### 2.5.5 Functional Annotation

In‐depth investigation of the functional aspects of microbes in skin lesions of different acne patients and comparison of the presence of functional differences between different groups requires subsequent functional annotation analysis. Phylogenetic Investigation of Communities by Reconstruction of Unobserved States (PICRUSt2) is a software that predicts functional abundance based solely on marker gene 16S sequences. It utilizes the tree of OTUs in the Greengene database and gene information on OTUs to infer the gene functional profiles of their common ancestors. Simultaneously, it infers the gene functional profiles of unmeasured species in the Greengene database, constructing gene functional prediction profiles for the archaeal and bacterial domains. Finally, by “mapping” the sequenced microbial composition to the database, microbial metabolic function prediction can be accomplished.

### 2.6 Cross‐Homology Correlation Network Analysis

Pearson's statistic was employed to calculate correlation coefficients between the relative abundance of each genus at the genus level and the quantitative values of different differential metabolites in patients with TAO. Subsequently, a heatmap of the correlation analysis was generated using R3.5.0.

## 3 Result

### 3.1 General Information

A total of 90 subjects were included in this study, including 30 patients with facial and back acne, 30 normal controls, and half male and half female in each group. There was no significant difference in age, gender, and acne severity between the two groups of patients with acne (*p* > 0.05) (Table 1).

**TABLE 1:** Clinical information of subjects.

	Control group	Facial acne group	Back acne group	*p*
Number of people	30	30	30	
Gender Male/Female	15/15	15/15	15/15	
Age	21.07 ± 2.664	21.7 ± 2.83	20.57 ± 1.547	0.4184
Acne grading				
I grade		21	17	0.6062
II grade		7	10	
III grade		2	3	

The analysis of the skin moisture content, trans epidermal water loss (TEWL), skin oil content, and skin pH of each group of subjects showed that compared with the normal control group, the facial TEWL and facial skin oil content in the facial acne group significantly increased, while the skin pH of the face and back significantly decreased; compared with the normal control group, the facial TEWL, facial and back skin oil content in the back acne group were significantly increased, while the facial and back skin pH was significantly decreased; compared with the facial acne group, the back acne group had significantly lower moisture content in the back skin and significantly higher oil content in the back skin. There was no significant difference in anxiety and depression status among the groups. The results of skin quality of life showed that compared with the normal control group, the score of quality of life in the back acne group increased significantly, indicating that back acne had a greater impact on the life of the subjects (Table 2).

**TABLE 2:** Skin condition, anxiety and depression status, and quality of life of the study subjects.

		Control group	Facial acne group	Back acne group	*p*
Skin moisture content	Face	64.63 ± 8.752	65.06 ± 8.158	60.67 ± 9.095	0.1028
	Back	34.92 ± 6.141	36.22 ± 7.287	31.35 ± 6.42△	0.0159
TEWL	Face	20.78 ± 3.554	24.8 ± 4.703*	25.78 ± 5.774#	0.0002
	Back	13.51 ± 3.545	14.21 ± 3.949	15.62 ± 2.326#	0.0401
Skin oil content	Face	74.6 ± 30.15	136.3 ± 43.41*	151.9 ± 37.3#	< 0.0001
	Back	19.43 ± 10.34	23.2 ± 11.36	32.08 ± 11.05△#	0.0001
Skin PH	Face	5.592 ± 0.4126	5.161 ± 0.4611*	5.147 ± 0.2852#	0.0001
	Back	5.494 ± 0.2914	5.091 ± 0.3064*	5.032 ± 0.2697#	< 0.0001
Anxiety and depression		4.667 ± 4.708	7.433 ± 7.257	6.767 ± 7.713	0.4462
Quality of life		5.5 ± 4.075	8.2 ± 4.992	10.07 ± 6.181#	0.0038

*Note:* * indicates that B is significantly different from A, with a *p* value of < 0.05, # indicates that C is significantly different from A, with a *p* value of < 0.05, and △ indicates that C is significantly different from B, with a *p* value of < 0.05.

### 3.2 Description of Sequencing Results

#### 3.2.1 OTU Count

**FIGURE 1:**
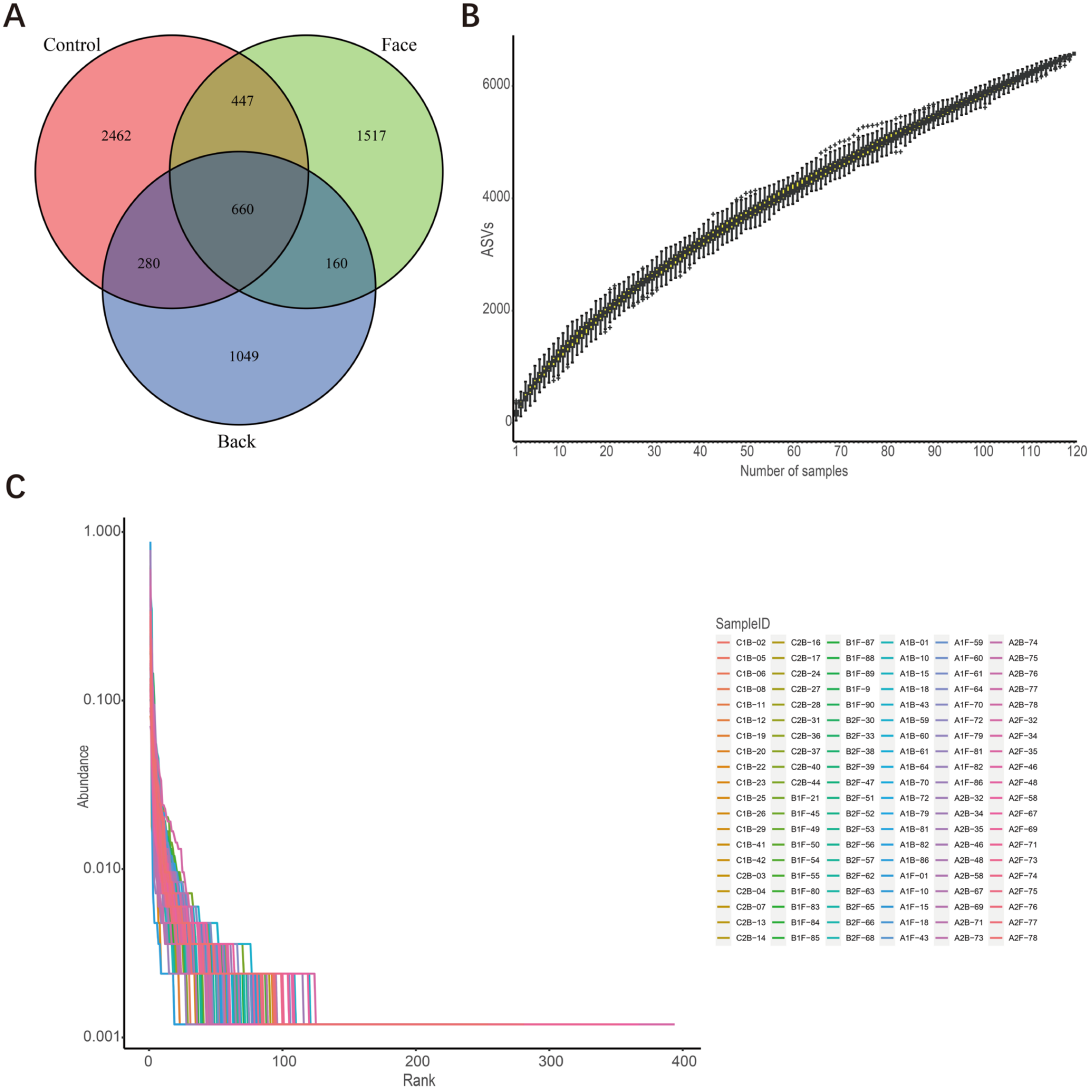
Description of sequencing results. (A) the Venn diagram results of OTU in the three groups; (B) species accumulation curve; (C) the abundance level curve of the three groups.

The Venn diagram results of this study showed that the total number of OTU in the three groups of samples was 6575, of which 660 were shared, 2462 were unique to the normal control group, 1517 were unique to the facial acne group, 1049 were unique to the back acne group, 477 were shared between the normal control group and the facial acne group, 280 were shared between the normal control group and the back acne group, and 160 were shared between the facial acne group and the back acne group (Figure 1A).

#### 3.2.2 Species Analysis

By plotting the species accumulation curve, it was found that as the sample size increased, the species accumulation curve rose steadily, indicating that the number of observed OTUs did not significantly increase with the increase in sample size, proving that the sample size in this study was sufficient and that further increases in sample size would not result in the discovery of new OTUs (Figure 1B).

#### 3.2.3 Abundance Level Curve

By plotting the abundance level curve, it was found that the curves of the three groups of samples had a large span, a smooth shape, and a slow decline, indicating that the species richness, diversity, and evenness of the three groups of samples were high (Figure 1C).

### 3.3 Analysis of Microbial Diversity

#### 3.3.1 Alpha Diversity Analysis

**FIGURE 2:**
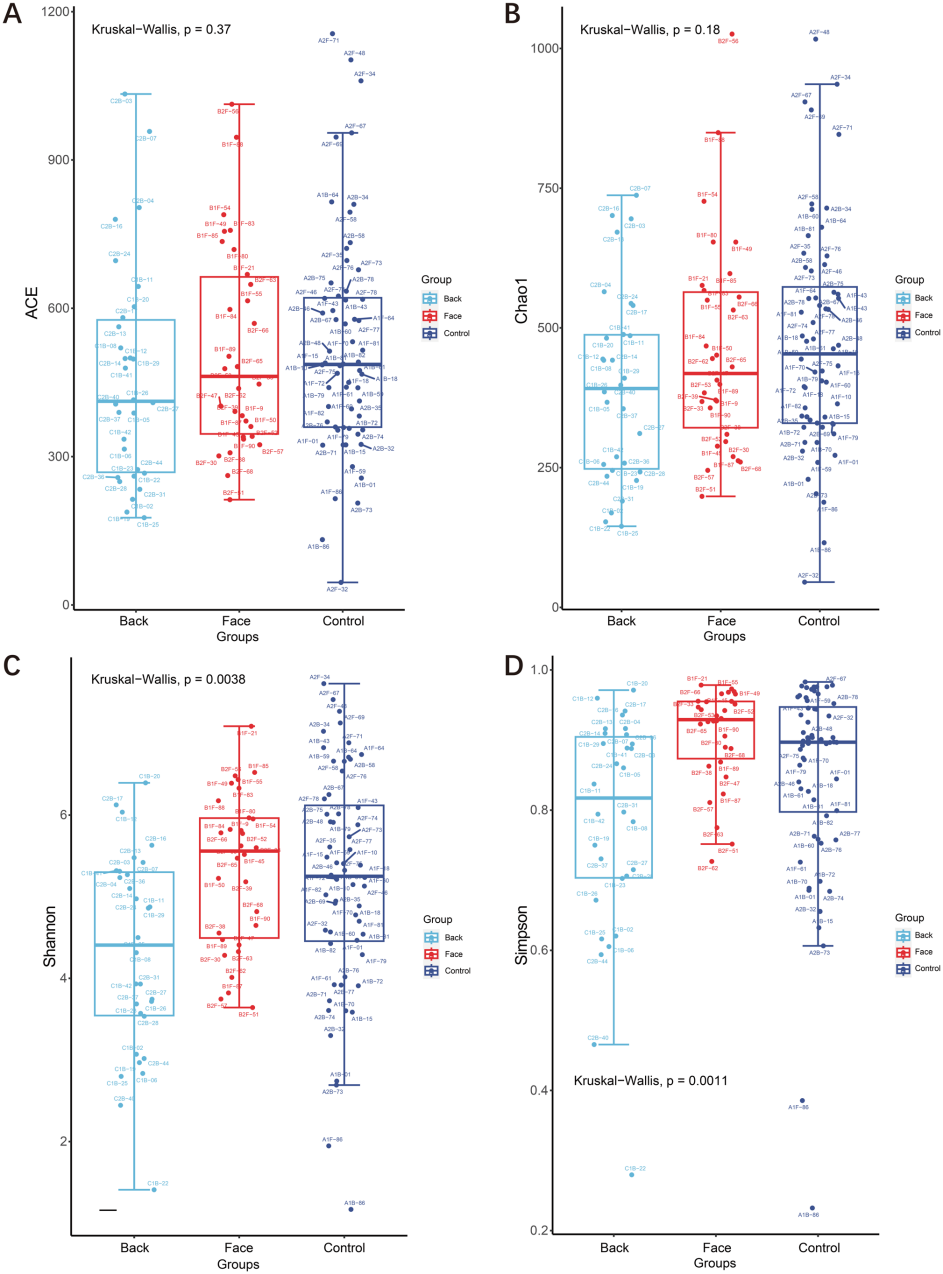
Three groups Alpha diversity ACE, Chao, Shannon index, and Simpson index.

According to the ACE and Chao indices, the species richness in the normal control group was slightly higher than that in the facial acne group and the back acne group, but there was no significant difference in species richness among the three groups (*p* > 0.05) (Figure 2A,B); according to the Shannon index and Simpson index, it was found that the species richness in the facial acne group was slightly higher than that in the normal control group and the back acne group, and there was a significant difference in species diversity among the three groups (*p* < 0.05) (Figure 2C,D). The species richness of the back acne group was the lowest among the four indices, indicating significant changes in the types of bacteria in patients with back acne.

#### 3.3.2 Beta Diversity Analysis

**FIGURE 3:**
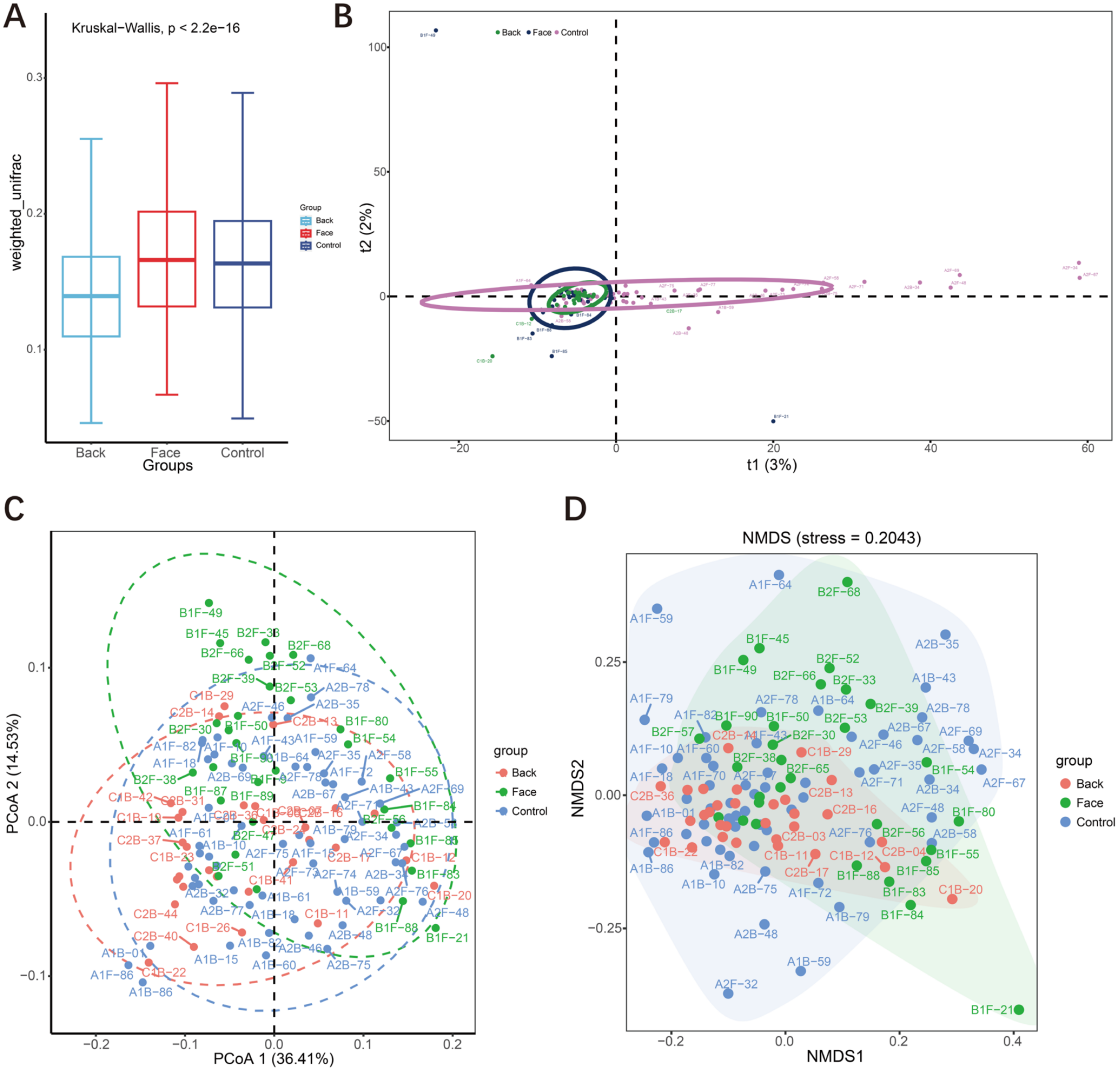
Beta diversity analysis. (A) the distance matrix calculated using the weighted unifrac method; (B) PCA analysis; (C) the PCoA utilizing the Weighted UniFrac distance metric; (D) the Bray‐Curtis distance for NMDS analysis.

This study drew a box plot of the differences in beta diversity between groups based on the distance matrix calculated using the weighted UniFrac method. It was found that the microbial community diversity was highest in the facial acne group, followed by the normal control group, and the microbial community diversity was lowest in the back acne group. The differences in microbial community diversity among the three groups were statistically significant (*p* < 0.01) (Figure 3A). Based on Principal Component Analysis (PCA) analysis, it was found that the bacterial flora in the normal control group was widely distributed, with a significantly reduced range of facial bacterial flora, concentrated on the left side of the X axis. The bacterial flora distribution in the back acne group was more concentrated than that in the facial acne group, suggesting that there may be differences in beta diversity among the three groups (Figure 3B). The PCoA utilizing the Weighted UniFrac distance metric revealed that the first two principal coordinates accounted for 50.94% of the variance in the skin microbial community composition. Specifically, axis I contributed 36.41% and axis II contributed 14.53% to the observed differences in community structure. The PCoA diagram shows that the normal control group and the back acne group are close to each other and have a lot of overlap. The back acne group is mainly located in the upper left corner, suggesting that there may be differences in beta diversity among the three groups (Figure 3C). Based on the Bray‐Curtis distance for NMDS analysis, the bacterial community distribution in the normal control group was widespread, while the facial bacterial community was slightly smaller. The back acne group was concentrated in a small area at the center of the image, suggesting that there may be differences in beta diversity among the three groups. We found that Stress = 0.2043. In the context of two‐dimensional Non‐metric Multidimensional Scaling (NMDS) analysis, it is widely accepted that a Stress value of less than 0.2 suggests a certain degree of explanatory significance. This indicates that the variations in beta diversity among the three groups are not statistically significant (Figure 3D).

### 3.4 Screening of Differential Microbiota and Biomarkers

In this study, LEfSe analysis was conducted to identify significantly different species with Latent Dirichlet Allocation (LDA) scores greater than 2, which serve as statistically significant biomarkers. The results revealed that there were 111 differential microbiota in the normal control group, 59 in the facial microbiota group, and 25 in the back acne group. Among the significantly different species with LDA scores greater than 4, the normal control group had 2 species: *o_Lachnospirales* and *f_Lachnospiraceae*; the facial microbiota group had 10 species: *p_Firmicutes, p_Proteobacteria, c_Bacilli, c_Gammaproteobacteria, o_Lactobacillales, f_Streptococcaceae, g_Streptococcus, g_Methyloversatilis, f_Rhodocyclaceae*, and *o_Burkholderiales*; and the back acne group had 8 species: *g_Cutibacterium, f_Propionibacteriaceae, o_Propionibacteriales, c_Actinobacteria, p_Actinobacteriota, g_Sphingomonas, o_Sphingomonadales*, and *f_Sphingomonadaceae*.

**FIGURE 4:**
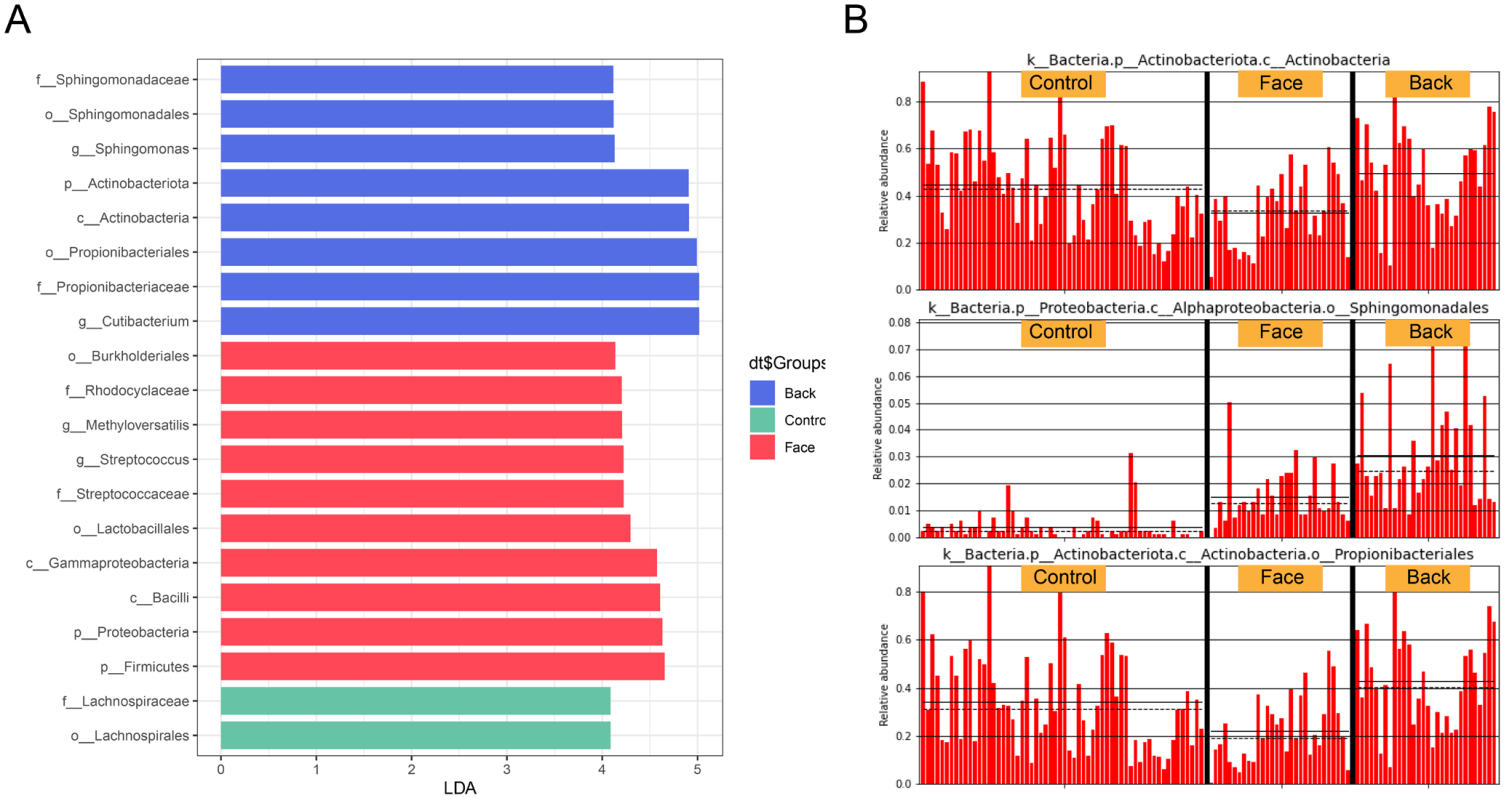
Difference analysis. (A) LEfSe analysis found species with LDA scores greater than 4; (B) Expression levels of three groups of representative species.

The STAMP differential analysis results showed that at the phylum level, the main differential microbiota included *Patescibacteria, Desulfobacterota, Proteobacteria, Verrucomicrobiota, Actinobacteriota, Fusobacteriota*, and *Firmicutes*; at the order level, there were 25 differential microbiota, including *Oceanospirillales, Sphingomonadales, Pseudonocardiales, Alteromonadales, Micrococcales, Desulfovibrionales*, and others. Based on the comprehensive results, it is suggested that *p_Actinobacteriota, o_Sphingomonadales*, and *o_Propionibacteriales* were all significantly increased in the back acne group (Figure 4).

### 3.5 Functional Annotation Analysis

**FIGURE 5:**
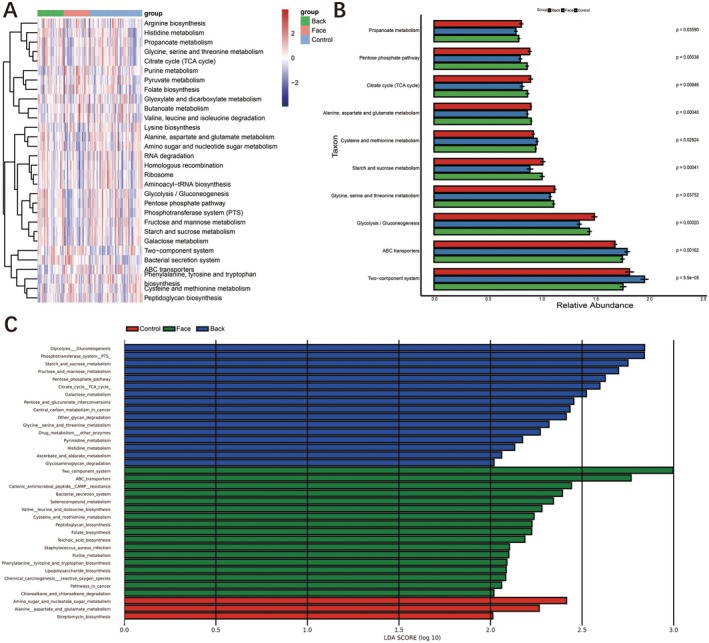
Function analysis KEGG.

Functional prediction analysis of the sequences from the three groups of samples was performed using PICRUSt analysis. The functional flora screened from the three groups of samples were mainly related to 36 Kyoto Encyclopedia of Genes and Genomes (KEGG) metabolic pathways. The specific KEGG pathways for the normal control group included amino sugar and nucleotide sugar metabolism, alanine aspartate and glutamate metabolism, etc. The specific KEGG pathways for the facial acne group included 17 pathways such as two‐component system, ABC transporters, etc. The specific KEGG pathways for the back acne group included 16 pathways such as glycolysis gluconeogenesis, phosphotransferase system PTS, starch and sucrose metabolism, fructose and mannose metabolism, pentose phosphate pathway, citrate cycle TCA cycle, galactose metabolism, etc. The functions of these pathways were primarily focused on carbohydrate metabolism (Figure 5).

**FIGURE 6:**
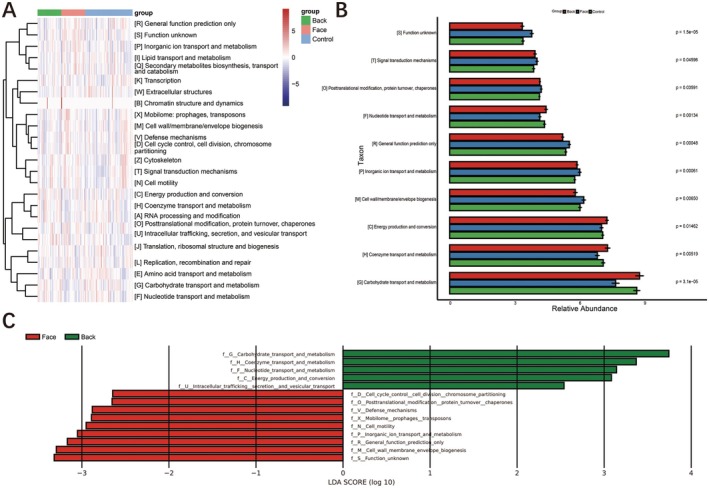
Function analysis GO.

The functional flora screened from the three groups of samples were mainly related to 14 Gene Ontology (GO) pathways. The specific GO for the facial acne group included 9 pathways such as Cell wall membrane envelope biogenesis, Inorganic ion transport and metabolism, General function prediction only, Posttranslational modification protein turnover chaperones, Function unknown, Defense mechanisms, Cell cycle control cell division chromosome partitioning, Cell motility, and Mobilome prophages transposons. The specific GO for the back acne group included 5 pathways such as Carbohydrate transport and metabolism, Coenzyme transport and metabolism, Energy production and conversion, Nucleotide transport and metabolism, Intracellular trafficking secretion and vesicular transport (Figure 6).

### 3.6 Correlation Analysis Between Microbiota and Skin Characteristics

**FIGURE 7:**
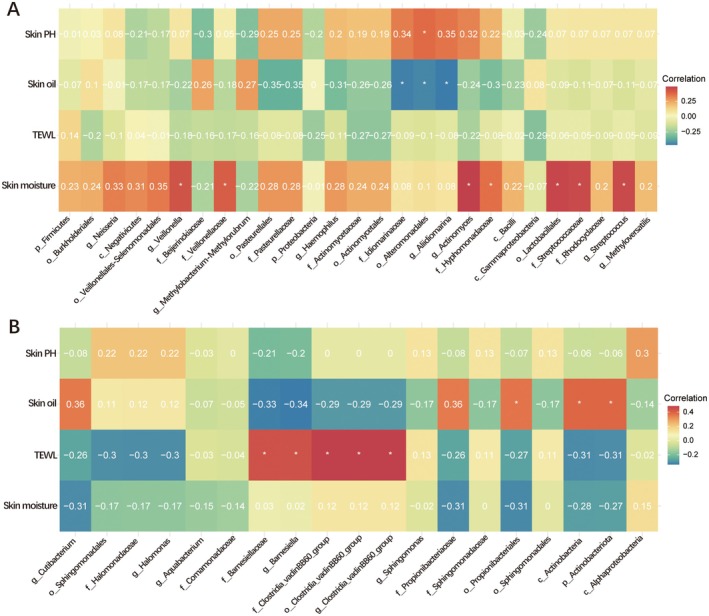
Correlation heatmap between differential microbiota and skin features: (A) facial acne, (B) back acne. * indicates that the association is significant, Red represents positive correlation, blue represents negative correlation.

The research results indicate a significant correlation between the composition of the skin microbiome and various skin parameters. In patients with facial acne, there is a positive correlation between the TEWL values of *o_Lactobacillales, f_Streptococcaceae, g_Streptococcus, g_Veillonella, f_Veillonellaceae, g_Actinomyces*, and *f_Hyphomonadaceae*. There is a negative correlation between the levels of *f_Idiomarinaceae, o_Alteromonadales*, and *g_Aliidiomarina* and skin oil content. There is a positive correlation between *o_Alteromonadales* and skin pH. In patients with acne on the back, *f_Barnesiellaceae, g_Barnesiella, f_Clostridia_vadinBB60_group, o_Clostridia_vadinBB60_group*, and *g_Clostridia_vadinBB60_group* are positively correlated with TEWL, while *o_Propionibacteriales, c_Actinobacteria*, and *p_Actinobacteriota* are positively correlated with skin oil content (Figure 7).

## 4 Discussion

Acne is a common skin condition that affects a large proportion of adolescents and young adults [11]. Acne develops due to factors like excess sebum, abnormal follicular keratinization, bacterial infection, and inflammation. The skin microbiota is crucial for maintaining the skin's barrier, protecting against harmful microorganisms. Skin‐associated microorganisms employ various mechanisms to resist these harmful entities, including resource competition, direct inhibition, and interference [12]. Recent evidence has increasingly highlighted the critical role of the skin microbiome in the pathogenesis of acne [13]. A healthy skin microbiome protects against pathogens and maintains balance, while major changes can lead to inflammation and worsen acne. Understanding the skin microbiome in acne sufferers is crucial for uncovering causes and guiding treatments.

In this study, we found that the diversity and richness of the skin microbiota in healthy individuals are significantly greater than those observed in acne patients. It suggests that healthy individuals tend to maintain a more balanced and diverse microbiota, whereas the microbiota composition in acne patients exhibits a marked ecological imbalance. The facial acne group showed higher diversity in the skin microbiome, as per the Shannon and Simpson indices, suggesting increased variability and potential disruption of microbial balance, possibly due to heightened responsiveness to environmental and host factors. In the beta diversity analysis, we found that the microbial communities within the normal control group exhibited greater diversity and a broader distribution range. In contrast, the microbial distribution in the acne groups was relatively concentrated, suggesting a notable structural disparity in the microbial communities between the two cohorts. This conclusion was further corroborated by PCoA and NMDS analyses, which revealed significant statistical differences in beta diversity metrics. This indicates that the richness and diversity of microbial communities in normal skin are crucial for maintaining skin health, whereas the microbial communities associated with acne display distinct and specific patterns [14].

Our LEfSe analysis identified distinct microbial genera in normal controls, facial acne, and truncal acne groups, suggesting a link between acne and changes in specific microbial populations. These genera may influence skin inflammation, sebum production, and immune responses, necessitating further study to clarify their roles in acne development. The facial acne group showed microbiota characteristics mainly composed of *p_Firmicutes*, *c_Bacilli*, *o_Lactobacillales*, *f_Streptococcae*, and *g_Streptococcus* et al. A previous clinical study showed that *p_Firmicutes* is relatively abundant in facial acne [15]. Streptococcus significantly contributes to facial acne, with high levels found in affected individuals. Acne improves with treatment as Streptococcus levels decrease [16]. The back acne group showed skin microbiota characteristics characterized by *g_Cutibacterium, f_Propionibacteriaceae, o_Propionibacteriales, c_Actinobacteria, p_Actinobacteriota* et al. Cutibacterium acnes (previously denominated *Propionibacterium acnes* ) is a component of the human microbiome found in several body districts [17], it is the main pathogenic microorganism involved in the formation of acne [18]. Cutibacterium acnes are important for maintaining skin homeostasis, as they can prevent pathogen colonization and help maintain skin PH [19]. However, the loss of microbial balance between Cutibacterium acnes subtypes can lead to acne [20]. Our study found that the abundance of Cutibacterium acnes in acne‐prone skin on the back was significantly higher than that in acne‐prone skin on the face, which is similar to previous research results [21].

In functional annotation analysis, predicted functional genes showed distinct traits across three groups. Acne patients' microbial communities displayed specific changes in metabolic pathways, particularly in antimicrobial response, metabolic shifts, and acid adaptation. Unique KEGG pathways in the facial acne group, like *Bacterial secretion system* and *Staphylococcus aureus infection*, suggest these may be crucial in facial acne pathogenesis. *Staphylococcus aureus* levels are higher in acne skin lesions and correlate with inflammation severity. Studies show that *Propionibacterium acnes* can worsen skin damage by increasing *Staphylococcus aureus* virulence and aiding its biofilm formation [22, 23]. Similarly, the truncal acne cohorts exhibited alterations in several distinct metabolic pathways, notably *Glycolysis/Gluconeogenesis*. Some strains of bacteria in the microbiota of acne on the back may rely on sugar metabolism pathways such as *Glycolysis/Gluconogenesis* to adapt to this environment, or affect the host's skin condition through metabolites. In addition, sugar metabolism involves a series of metabolic intermediates, such as lactate, which may locally trigger or exacerbate inflammatory reactions. Previous studies indicate that targeting the glycolysis pathway with medication can effectively treat acne by suppressing pathogenic skin bacteria [24]. These findings may offer novel insights into the relationship between shifts in the microbial community and the pathogenesis of acne.

We assessed back acne patients for hydration, TEWL, sebum production, and pH. Compared to facial acne patients, those with back acne had significantly lower moisture and higher oil content on their back skin. This study found that in patients with back acne, certain microbial groups (*f_Barnesiellaceae, g_Barnesiella, f_Clostridia_vadinBB60_group, o_Clostridia_vadinBB60_group*, and *g_Clostridia_vadinBB60_group*) are positively correlated with TEWL, while *o_Propionibacteriales, c_Actinobacteria*, and *p_Actinobacteriota* are positively correlated with skin oil content. It is speculated that the reason for this phenomenon may be related to the neglect of back care and the difference in the choice of cleaning agents for face and back care. Facial cleansers typically use milder surfactants like amino acids and betaine, while body washes opt for stronger surfactants like SLS and SLES due to the body's larger cleaning area and oil production. However, these stronger cleansers can damage the skin barrier in individuals with trunk acne, impacting oil secretion and moisture levels. The effect of surfactants on skin microbial abundance has also been confirmed in other studies [25].

This study has three key limitations. First, the small Beijing‐based sample limits generalizability, requiring multi‐regional validation. Second, while short‐term dietary habits were controlled, long‐term dietary patterns (e.g., sustained high‐glycemic/high‐fat intake) were not assessed, though such diets may cumulatively alter gut microbiota and subsequently affect skin ecosystems via chronic inflammation or lipid metabolism. Future studies should incorporate longitudinal dietary records (e.g., food frequency questionnaires) to disentangle these effects. Third, data collection restricted to April–June overlooks seasonal impacts like humidity/UV‐induced sebum fluctuations; multi‐season sampling is needed. Residual lifestyle confounders warrant advanced statistical modeling in follow‐up research.

## 5 Conclusion

In summary, healthy individuals have more diverse and richer skin microbiota, whereas acne patients experience significant ecological imbalance. There are notable differences in microbiota across acne sites, linked to variations in specific microbial populations. The microbiota in truncal acne patients mainly focuses on sugar metabolism and shows significant links with skin moisture and oil levels. These insights enhance understanding of acne pathogenesis and suggest new therapeutic approaches. For instance, our findings indicate that, in comparison to facial acne, truncal acne exhibits reduced microbial abundance and a more pronounced microecological imbalance. This disparity may be attributed to insufficient care for the trunk or the choice of surfactants in personal care products. These findings help to remind us that we need to be vigilant about the choice of surfactant components in body care products, especially for people with back acne. It is necessary to consider designing an effective formula to repair the ecological balance of the skin.

## Author Contributions

All authors made a significant contribution to the work reported, whether that is in the conception, study design, execution, acquisition of data, analysis, and interpretation, or in all these areas; took part in drafting, revising, or critically reviewing the article; gave final approval of the version to be published; have agreed on the journal to which the article has been submitted; and agree to be accountable for all aspects of the work.

## Ethics Statement

This observational retrospective study was conducted in accordance with the Declaration of Helsinki and approved by the Scientific Research Ethics Committee, Beijing Technology and Business University (No. 73, 2024).

## Conflicts of Interest

The authors declare no conflicts of interest.

## Data Availability

The data that support the findings of this study are available from the corresponding author upon reasonable request.
